# Mechanistic insights into ferroptosis and its therapeutic potential in hepatocellular carcinoma

**DOI:** 10.3389/fimmu.2026.1833582

**Published:** 2026-07-15

**Authors:** Fan Yang, Zhongjie Li, Bao Zheng

**Affiliations:** 1 Department of Clinical Laboratory, Shandong Provincial Hospital affiliated to Shandong First Medical University, Jinan, China; 2 Department of Clinical Laboratory, Shandong Second Provincial General Hospital, Jinan, China; 3 Department of Clinical Laboratory, Women and Children’s Hospital, Qingdao University, Qingdao, China

**Keywords:** epigenetic, ferroptosis, HCC, metabolic, microenvironmental, therapy

## Abstract

Ferroptosis is an iron-dependent form of programmed cell death driven by lipid peroxidation, distinct from apoptosis and necrosis. It has been confirmed as a key regulator in hepatocellular carcinoma (HCC). This study systematically elucidates three interconnected regulatory networks of ferroptosis—metabolic, epigenetic, and microenvironmental—and proposes a closed-loop regulatory model integrating these dimensions. By integrating metabolic biomarkers, epigenetic indicators, and microenvironmental features, we also summarize emerging strategies to enhance ferroptosis sensitivity. By summarizing the regulatory nodes as well as clinical translation progress, this work provides a comprehensive roadmap for overcoming therapeutic bottlenecks in HCC and realizing ferroptosis-based precision medicine.

## 1 Introduction

According to the GLOBOCAN 2022 global cancer statistics (1), hepatocellular carcinoma (HCC) ranks as the sixth most common malignant tumor and the third leading cause of cancer-related deaths worldwide. In 2022, over 860,000 new cases and more than 750,000 deaths were estimated globally, with China accounting for over 7.6% of the global incidence. Most patients are diagnosed at an advanced stage, and conventional treatments—including surgery, targeted therapy, and immunotherapy—face significant challenges due to high rates of drug resistance and recurrence. In recent years, ferroptosis, a form of non-apoptotic cell death driven by lipid peroxidation, has emerged as a promising novel therapeutic target in HCC due to its unique metabolic dependencies (2).

This article first systematically elucidates the core metabolic mechanisms, epigenetic regulation, and microenvironmental regulatory networks of ferroptosis in HCC, proposing an integrated metabolic–epigenetic–microenvironment closed-loop regulatory model. It then outlines the key regulatory nodes of ferroptosis in HCC, followed by an in-depth analysis of the clinical translation progress, toxicity risks, and core challenges associated with ferroptosis-targeted therapies. Finally, future key research directions in this field are identified.

## 2 Core metabolic mechanisms of ferroptosis

### 2.1 The double-edged sword effect of lipid peroxidation

Ferroptosis was first proposed as a novel concept in 2012 (3). It is an iron-dependent, non-apoptotic form of cell death characterized by the accumulation of lipid peroxides and reactive oxygen species (ROS). The core mechanism of ferroptosis lies in the dynamic balance between oxidative damage and antioxidant defense. The glutathione/glutathione peroxidase 4 (GSH/GPX4) axis is the central pathway inhibiting lipid peroxidation and preventing ferroptosis within this process (this will be systematically elaborated below; subsequent sections will focus on the specific regulatory roles within this pathway). The identification of its key pathways and targets has been a major research focus.

Oxidative damage in ferroptosis is primarily caused by lipid peroxidation. The initiation mechanisms of lipid peroxidation can be categorized into two types: enzymatic and non-enzymatic. Enzymes catalyzing lipid peroxidation are mostly heme or non-heme iron enzymes. Among them, enzymes of the arachidonate lipoxygenase (ALOX) family belong to lipoxygenases, whose active sites contain non-heme iron. These enzymes can directly catalyze the formation of lipid radicals, triggering oxidative cascade reactions (4). Long-chain acyl-CoA synthetase 4 (ACSL4) and lysophosphatidylcholine acyltransferase 3 (LPCAT3) are involved in activating arachidonic acid, which can also form lipid radicals and promote ferroptosis (5). Non-enzymatic lipid peroxidation is initiated by redox-active metals, particularly iron. Iron ions propagate lipid peroxidation via the Fenton reaction (6). Studies have also found that lipid peroxides formed by iron enzymes can serve as substrates for the Fenton reaction (4).

The antioxidant enzyme glutathione peroxidase 4 (GPX4) is a core inhibitor of ferroptosis. It suppresses the accumulation of lipid peroxides, thereby preventing ferroptosis (7). Glutathione (GSH) enhances the activity of GPX4, forming the GSH/GPX4 axis, which is currently the primary pathway for inhibiting lipid peroxidation and preventing ferroptosis. The synthesis of GSH is regulated by the cystine/glutamate antiporter (system x_c^–^). System x_c^–^ is an amino acid antiporter located on the cell membrane, primarily composed of solute carrier family 7 member 11 (SLC7A11) and solute carrier family 3 member 2 (SLC3A2). It mediates the uptake of cystine, which is subsequently reduced to cysteine for GSH synthesis (8). It has been reported that nuclear factor erythroid 2-related factor 2 (NRF2) acts on SLC7A11, promoting GSH synthesis and exerting a positive anti-ferroptosis effect; however, in-depth research from this perspective remains insufficient.

### 2.2 Dynamic homeostasis of iron metabolism

Iron is an essential trace element in the human body, with an adult content of approximately 3-5 g. Dietary intake serves as the source of iron, the majority of which is stored in the liver, muscle tissue, and red blood cells (9). The entry of iron into the bloodstream primarily relies on transport by transferrin (TF). Transferrin first binds iron, and the iron-bound transferrin is then transported into intracellular vesicles called endosomes via transferrin receptor (TFRC)-mediated endocytosis. Iron is subsequently released from the endosomes with the assistance of divalent metal transporter 1 (DMT1) (10). The released iron is utilized for hemoglobin synthesis in the bone marrow and myoglobin synthesis in muscle tissue. Excess iron is stored within cells by binding to ferritin (11). Studies have shown that reducing systemic iron levels can inhibit ferroptosis. The adaptor protein nuclear receptor coactivator 4 (NCOA4) mediates ferritinophagy, leading to the release of intracellular iron and consequently increasing sensitivity to ferroptosis (12).

Ferritinophagy refers to the process by which cells degrade their primary iron storage protein, ferritin, via the autophagy pathway. This process leads to the release of intracellularly stored iron, thereby increasing the content of the cellular “labile iron pool” (LIP). Ferritin serves as the main intracellular storage form of iron, sequestering it to prevent the catalysis of harmful oxidative reactions. Ferritinophagy degrades ferritin through the autophagic–lysosomal pathway, releasing a substantial amount of iron ions, which directly increases the concentration of labile iron in the cytosol. The labile iron pool is a key catalyst that triggers the Fenton reaction, initiating and amplifying the chain reaction of lipid peroxidation (13). Consequently, by elevating labile iron levels, ferritinophagy significantly enhances cellular susceptibility to ferroptosis.

### 2.3 Synergistic action of the antioxidant system

As a GPX4-independent pathway, ferroptosis suppressor protein 1 (FSP1)-CoQ10 also plays a crucial role in regulating ferroptosis. FSP1 is an NAD(P)H-dependent oxidoreductase. At the plasma membrane, FSP1 reduces coenzyme Q10 (CoQ10, ubiquinone) to ubiquinol (CoQ10H2). Ubiquinol acts as a lipophilic radical-trapping antioxidant, capturing free radicals generated during lipid peroxidation, thereby blocking the lipid peroxidation chain reaction and preventing membrane damage (14). This mechanism ultimately suppresses ferroptosis.

The GCH1–BH4–DHFR axis inhibits ferroptosis. The guanosine triphosphate (GTP) cyclohydrolase-1 (GCH1)–tetrahydrobiopterin (BH4) pathway also represents a GPX4-independent regulatory pathway for ferroptosis. GTP cyclohydrolase 1 (GCH1) regulates the synthesis of BH4, which exerts a protective effect by reducing CoQ10 to capture free radicals (15).

Dihydroorotate dehydrogenase (DHODH) is an enzyme located in the inner mitochondrial membrane, constituting a ferroptosis defense system independent of cytosolic GPX4 and plasma membrane FSP1. It specifically inhibits lipid peroxidation within mitochondria by catalyzing the reduction of CoQ to the antioxidant CoQH2, thereby preventing ferroptosis. When GPX4 function is impaired or SLC7A11 expression is low, cellular dependence on DHODH is enhanced. DHODH deficiency or inhibition leads to a decrease in mitochondrial CoQH2 levels and an accumulation of reactive oxygen species (ROS), consequently promoting mitochondrial lipid peroxidation and the onset of ferroptosis (16).

### 2.4 Lipid metabolism reprogramming and dietary effects

The novel concept of “dietary effects on ferroptosis” was published in *Nature*. This study demonstrated for the first time that the ratio of polyunsaturated fatty acids (PUFAs) to monounsaturated fatty acids (MUFAs) in the diet is a key physiological factor determining the sensitivity of T cells to ferroptosis. It proved that ferroptosis is regulated not only by endogenous cellular genes but also directly by exogenous nutrient intake (dietary lipid composition). Furthermore, the molecular pathway through which dietary signals are converted into cell death signals was elucidated: long-chain acyl-CoA synthetase 4 (ACSL4) is highly expressed in T cells, where it preferentially catalyzes the activation of PUFAs and incorporates them into membrane phospholipids. ACSL4 not only establishes the lipid substrate foundation for ferroptosis but also serves as the core “molecular switch” linking external dietary lipids to the fate of internal T cells (17).

## 3 Epigenetic regulations

The expression and activity of key molecules within the core ferroptosis metabolic pathways can be precisely regulated at the epigenetic level. The sponge effect of non-coding RNAs and posttranscriptional RNA modifications can influence the sensitivity of hepatoma cells to ferroptosis by regulating metabolism-related targets, serving as a crucial link between metabolism and microenvironmental regulation.

### 3.1 Expression of key non-coding RNAs

Nuclear paraspeckle assembly transcript 1 (NEAT1) is a long non-coding RNA (lncRNA) that functions as a competing endogenous RNA (ceRNA), acting as a molecular sponge for various microRNAs (miRNAs) (18, 19). It is associated with tumor proliferation, invasion, survival, drug resistance, and metastasis (20). Studies have shown that in hepatoma cells, NEAT1 competitively binds to miR-362-3p, alleviating the inhibitory effect of miR-362-3p on myo-inositol oxygenase (MIOX) mRNA, thereby upregulating the MIOX expression. Subsequently, MIOX promotes reactive oxygen species (ROS) production, reduces the levels of reduced nicotinamide adenine dinucleotide phosphate (NADPH) and glutathione (GSH), enhances lipid peroxidation, and ultimately exacerbates ferroptosis (21).

### 3.2 Posttranscriptional RNA modifications

In the context of posttranscriptional RNA modifications, various proteins function as “readers,” “writers,” and “erasers” of N6-methyladenosine (m6A) to regulate mRNA metabolism (22). thereby influencing the occurrence of ferroptosis in cancer. In HCC, m6A modification mediated by the “writer” methyltransferase-like protein 3 (METTL3) is generally oncogenic (23). Ferroptosis suppressor protein 1 (FSP1) is a key inhibitor of ferroptosis, and its expression is regulated by multiple layers of m6A epigenetic modifications. Research indicates that METTL3 can lead to decreased FSP1 mRNA levels in patients with aortic dissection (24). In lung cancer, the “reader” protein YTH domain-containing protein 1 (YTHDC1) also suppresses FSP1 protein expression (25). In colorectal cancer, the “eraser” protein fat and obesity-associated protein (FTO) promote FSP1 mRNA degradation (26), whereas FSP1 inhibits ferroptosis in HCC (14, 27). Although the non-specific hepatocellular carcinoma model cannot yet provide direct evidence, the role of ferroptosis suppressor protein 1 (FSP1) as a common target in inhibiting ferroptosis in hepatocellular carcinoma remains a key focus of our subsequent investigations.

## 4 Microenvironmental regulation

The tumor microenvironment is not an independent layer in the regulation of ferroptosis; it forms bidirectional interactions with metabolic and epigenetic regulation through mechanisms such as mechanical and immune signaling. The mechanical properties of the microenvironment can directly regulate the expression of key metabolic enzymes, whereas the release of metabolites triggered by ferroptosis can reshape the immune status of the microenvironment. These three components constitute a self-reinforcing regulatory network for ferroptosis in hepatocellular carcinoma (HCC).

### 4.1 Microenvironmental feedback

The extracellular matrix (ECM) plays a crucial role in cellular function. The ECM not only serves as a scaffold supporting cells but also influences cellular function by modulating the cellular niche (28). Changes in the ECM, which occur when cells perform different functions, are thought to be related to matrix stiffness. Integrin β1 connects cells to the ECM, facilitating bidirectional signal transduction (29). The expression of integrins increases with elevated matrix stiffness. When matrix stiffness rises above a certain threshold, structural proteins such as focal adhesion kinase (FAK) and Yes-associated protein (YAP) are activated by integrins (30), promoting the growth and invasion of various cancer cells, including HCC cells (31). Acyl-CoA synthetase long-chain family member 4 (ACSL4) is a key enzyme promoting ferroptosis in HCC cells. When upstream integrin signaling is inhibited, the expression of ACSL4 is correspondingly reduced, thereby suppressing ferroptosis (32).

The regulatory axis of matrix stiffness → Integrin β1/FAK/YAP signaling → ACSL4 upregulation untangles the molecular logic by which hepatoma cells convert microenvironmental stimuli into ferroptosis sensitivity through mechanotransduction. However, in certain contexts of acute injury or specific tumor microenvironments, YAP activation may not aim to induce cell death. Instead, it can serve as a stress response, protecting tissues from excessive damage caused by ferroptosis through the “negative regulatory” function of NEDD4L (33). This provides a more nuanced therapeutic direction for modulating YAP activity.

### 4.2 Release of microenvironmental death signals and immune amplification

Ferroptosis is initiated by lipid peroxidation leading to plasma membrane rupture. Upon membrane disruption, intracellular damage-associated molecular patterns (DAMPs) are passively released into the extracellular microenvironment. The release of death signals occurs via three primary mechanisms (1). Exposure of calreticulin (CRT): During the early stages of ferroptosis, CRT translocates from the endoplasmic reticulum to the cell surface, where it is directly recognized by dendritic cells (DCs) (34) (2). Release of adenosine triphosphate (ATP) as a chemoattractant: When cells rupture during death, ATP leaks from the intracellular to the extracellular space. Extracellular ATP attracts macrophages and natural killer (NK) cells via the P2X7 receptor and promotes DC maturation (34) (3). Passive release of high-mobility group box 1 (HMGB1): Following cell lysis, nuclear HMGB1 is released extracellularly along with nuclear fragments. By binding to Toll-like receptor 4 (TLR4), HMGB1 induces DC maturation and enhances their antigen-presenting capacity (35).

Following the release of these death signals, the microenvironment undergoes a cascade of reactions leading to large-scale activation and infiltration of immune cells. The first step is DC activation: DAMPs stimulate DCs to migrate to lymph nodes, present tumor antigens, and activate CD8+ T cells (36). This is followed by macrophage polarization: Lipid peroxides can stimulate the polarization of macrophages toward the M1 phenotype (37).

However, there are significant differences in the regulatory mechanisms between tumor cells and immune cells. Ferroptosis in tumor cells depends on the accumulation of lipid peroxidation and reactive oxygen species, whereas in immune cells, cytokines such as interferon-gamma (IFNγ) typically downregulate the expression of system x_c^–^, reduce GSH synthesis, and thereby increase their sensitivity to ferroptosis (38).

Ferroptosis is not an instantaneous event but rather a dynamic process. As intracellular metabolic disorders progress, cells undergo a staged transition from “self-rescue” to “collapse”. In the early stage, intracellular glutathione (GSH) depletion and decreased GPX4 activity lead to the rapid accumulation of lipid peroxides (LPO) (39), accompanied by typical morphological changes, including the loss of mitochondrial cristae, reduced mitochondrial volume, and decreased membrane potential (15). When the cellular defense mechanisms are completely overwhelmed, the cell enters an irreversible “death” state, characterized by cell membrane rupture and the release of large amounts of damage-associated molecular patterns (such as HMGB1 and ATP) (39), along with the disintegration of cellular organelles.

Oxidized lipids play a “double-edged sword” role in the tumor microenvironment. Although they serve as executors of ferroptosis, when taken up by immune cells, they often lead to immune system dysfunction. First, T-cell function is inhibited: activated T cells in the tumor microenvironment abnormally upregulate CD36 expression. Through CD36, T cells absorb these harmful substances in large quantities (40), resulting in decreased proliferative capacity and significantly impaired antitumor activity (41). Second, dendritic cell maturation is blocked: oxidized phospholipids such as Ox-PAPC bind to receptors (CD14/MD2) on the surface of dendritic cells, competitively interfering with normal pathogen recognition signals, thereby inhibiting dendritic cell maturation and antigen presentation capabilities (42).

Not only in hepatocellular carcinoma but also in the pathogenesis of liver diseases, chronic hepatitis, fibrosis, and cirrhosis progressively remodel the hepatic immune microenvironment. During the chronic hepatitis phase, persistent viral or toxic stimuli induce oxidative stress and iron accumulation, promoting hepatocyte death and the release of damage-associated molecular patterns (DAMPs), which further recruit immune cells (43), exacerbating tissue damage and triggering the fibrotic process. Activated hepatic stellate cells (HSCs) are the primary executors of fibrosis, and inducing ferroptosis in HSCs can block fibrosis progression (44). In advanced cirrhosis, the immune system is often in an exhausted or suppressed state, rendering it unable to effectively utilize antigens generated by ferroptosis for immune surveillance (45).

## 5 The metabolism–epigenetics–microenvironment circuit

**Figure 1:**
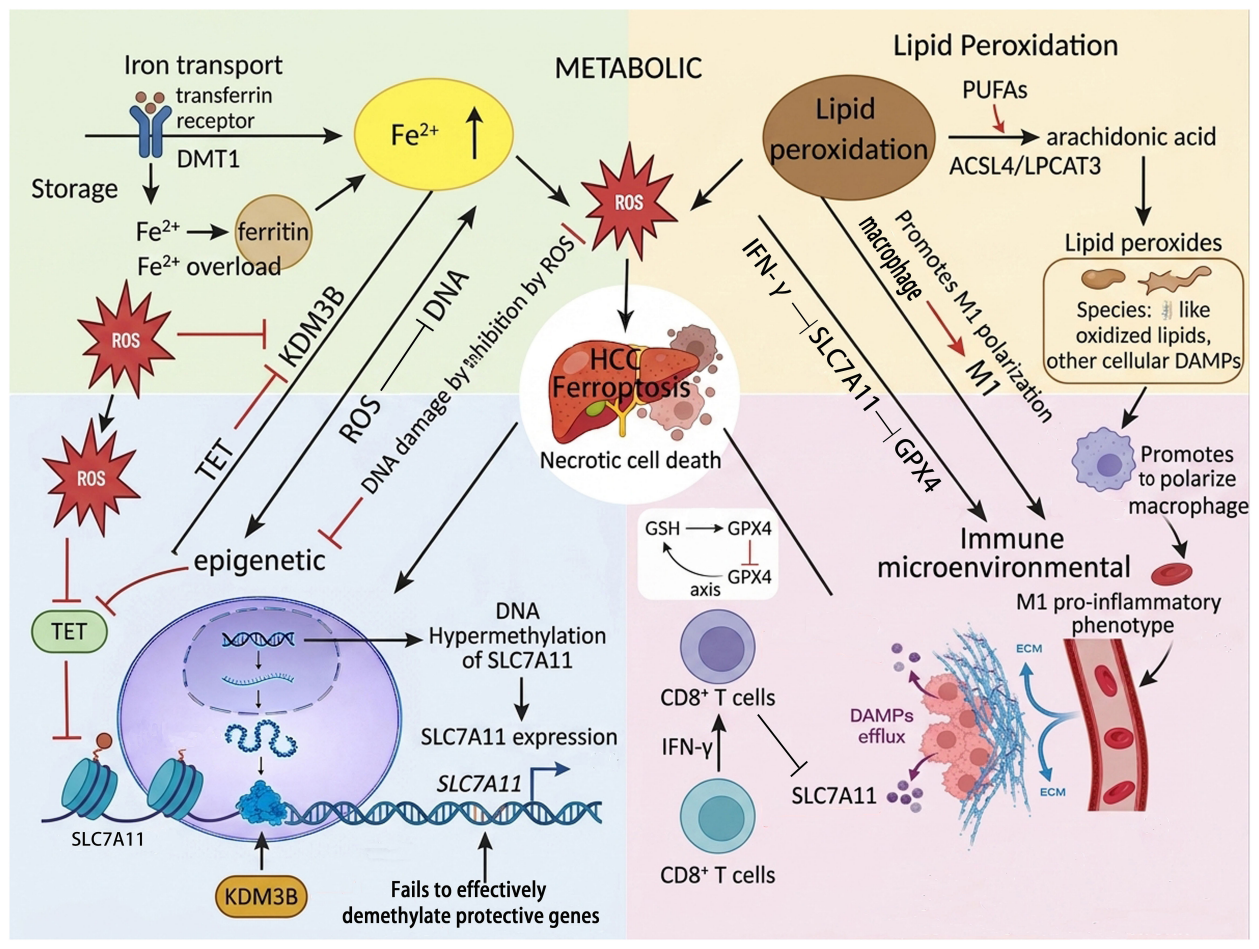
Schematic model of ferroptosis and its epigenetic and immune regulation in HCC. Iron transport and Fe^2+^ overload induce ROS generation and lipid peroxidation (mediated by ACSL4/LPCAT3). Epigenetic modifications (KDM3B/TET-mediated SLC7A11 hypermethylation) inhibit the GPX4/GSH antioxidant axis, leading to ferroptosis and necrotic cell death. The release of DAMPs and oxidized lipids promotes M1 macrophage polarization via IFN-γ, thereby disrupting the defensive tumor microenvironment (involving CD8+ T cells and ECM).

In summary, ferroptosis in hepatocellular carcinoma (HCC) results from the interplay of metabolic dysregulation, epigenetic control, and the tumor microenvironment. These mechanisms do not operate in isolation but interact through specific biological circuits, forming a self-reinforcing lethal network (**Figure 1**). Metabolism drives epigenetics: Iron overload induces reactive oxygen species (ROS) that directly damage DNA and inhibit the activity of ten-eleven translocation (TET) demethylases, thereby promoting the methylation of SLC7A11 (46). Epigenetics regulates metabolism: ROS-induced damage inhibits TET enzymes, leading to DNA hypermethylation. Consequently, KDM3B fails to effectively demethylate protective genes, further promoting ferroptosis (47). Metabolism induces immune responses: Lipid peroxides are released as damage-associated molecular patterns (DAMPs), stimulating macrophage polarization toward the M1 phenotype and disrupting the defensive tumor immune microenvironment (48). Immune responses amplify metabolism: CD8^+^ T cells secrete IFN-γ, which suppresses glutathione peroxidase 4 (GPX4) activity by downregulating the expression of system x_c^–^, reducing GSH synthesis, and further exacerbating ferroptosis (49) (for details on the GSH/GPX4 axis mechanism, see Section 1.1).

Although systems integration is the prevailing trend in current ferroptosis research, a significant limitation lies in the insufficiency of the chain of the system. Direct evidence for certain specific molecular mechanisms—such as the inhibition of TET and KDM3B—remains relatively weak in the context of HCC due to the highly heterogeneous histological characteristics of hepatocellular carcinoma and the difficulty in obtaining high-quality biopsy samples from patients. Many studies involving epigenetic regulation require fresh tissue for sequencing. Consequently, many inferences are based on analogies from other cancer types. Similarly, while the mechanism by which IFN-γ suppresses GPX4 is widely discussed in tumor immunology, in HCC, IFN-γ does not solely inhibit GPX4 by downregulating the expression of system x_c^–^. Conversely, some studies have found that IFN-γ exposure can upregulate GPX4 expression through the regulation of liver cancer stem cells (LCSCs) in HCC (50). However, this may represent a tumor cell resistance response to IFN-γ, leading to enhanced ferroptosis resistance mediated by GPX4.

## 6 Strategies to enhance ferroptosis sensitivity

**Figure 2:**
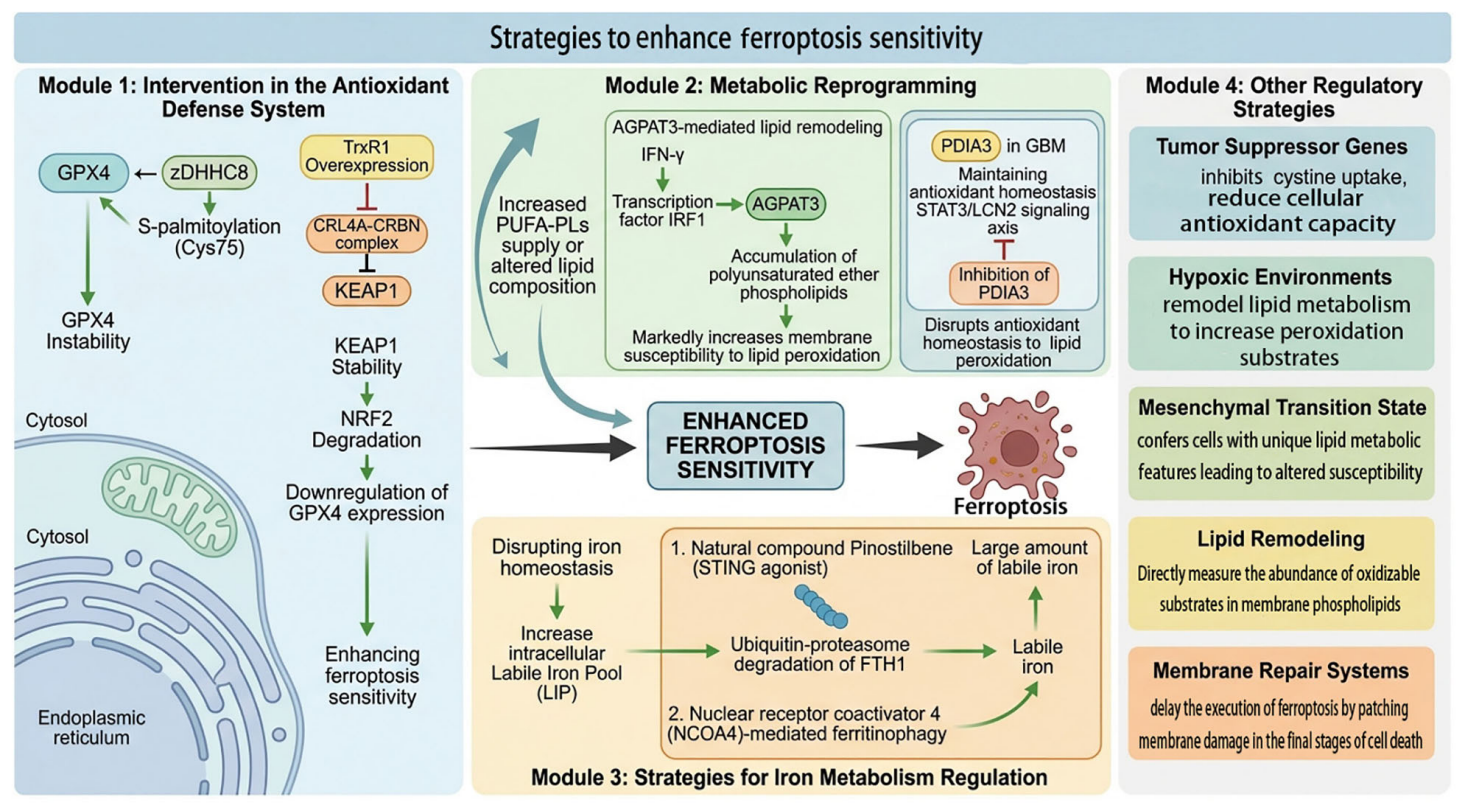
Strategies to enhance ferroptosis sensitivity. Module 1 targets the antioxidant defense system by downregulating GPX4 and disrupting antioxidant homeostasis via the CRL4A–CRBN complex and zDHHC8 S-palmitoylation. Module 2 reprograms lipid metabolism, where AGPAT3 overexpression drives PUFA-PL accumulation to increase peroxidation susceptibility. PDIA3 maintains the antioxidant homeostasis of glioblastoma via the STAT3/LCN2 signaling axis. Module 3 regulates iron metabolism by expanding the labile iron pool via NCOA4-mediated ferritinophagy and pharmacological disruption (e.g., pinostilbene and STING agonists). Module 4 involves other regulatory mechanisms, including hypoxic responses, mesenchymal transition state, lipid remodeling, membrane repair systems, and tumor suppressor gene activation.

The above sections describe the molecular mechanisms by which ferroptosis functions in the hepatocellular carcinoma (HCC) microenvironment and its potential therapeutic targets. Research has found that enhancing ferroptosis sensitivity has also become an important approach in treating other diseases (**Figure 2**). These strategies may similarly represent potential targets for HCC therapy.

### 6.1 Intervention in the antioxidant defense system

Cells possess multiple layers of antioxidant defense. Inhibiting these defenses can directly or synergistically induce ferroptosis. Studies have found that zinc finger DHHC-domain containing protein 8 (zDHHC8) mediates S-palmitoylation of GPX4 at the Cys75 site, which is crucial for GPX4 stability and its anti-ferroptosis function. Inhibition of zDHHC8 leads to GPX4 instability, thereby enhancing ferroptosis sensitivity (51). Another study demonstrated that in various cancer cells, thioredoxin reductase 1 (TrxR1) has a dual regulatory role: its overexpression can inhibit the CRL4A-CRBN complex-mediated degradation of KEAP1, enhance KEAP1 stability, subsequently promote NRF2 degradation, downregulate GPX4 expression, and consequently enhance ferroptosis sensitivity (52). Therefore, it is reasonable to hypothesize that a pathway promoting ferroptosis via TrxR1 may also exist in HCC.

### 6.2 Metabolic reprogramming

The essence of ferroptosis is lipid peroxidation. Sensitivity to ferroptosis can be significantly enhanced by increasing substrate (PUFA-PLs) supply or altering lipid composition. One study found that AGPAT3-mediated lipid remodeling activates the transcription factor IRF1 via IFN-γ, upregulating AGPAT3 expression. AGPAT3 drives lipid remodeling, leading to the accumulation of polyunsaturated ether phospholipids, which markedly increases the susceptibility of tumor cell membranes to lipid peroxidation and enhances the efficacy of immune checkpoint blockers (ICBs) (53). Similarly, in glioblastoma (GBM), PDIA3 maintains the antioxidant homeostasis of glioblastoma via the STAT3/LCN2 signaling axis. Inhibition of PDIA3 disrupts this homeostasis and significantly increases the sensitivity of GBM cells to ferroptosis inducers (54).

### 6.3 Strategies for iron metabolism regulation

Iron is a core element catalyzing lipid peroxidation. Disrupting iron homeostasis to increase the intracellular labile iron pool (LIP) can enhance susceptibility to ferroptosis. Research has found that in lung cancer, the natural compound pinostilbene, acting as a novel STING agonist, induces the degradation of ferritin heavy chain (FTH1) via the ubiquitin–proteasome system, releasing a large amount of labile iron and significantly enhancing the sensitivity of lung cancer cells to ferroptosis (55). Similarly, nuclear receptor coactivator 4 (NCOA4)-mediated ferritinophagy can also increase the risk of ferroptosis by releasing labile iron (56).

### 6.4 Other regulatory strategies

As a complex form of cell death, the regulatory network of ferroptosis exhibits high diversity. Beyond the classical antioxidant metabolic pathways, multiple biological processes can influence the sensitivity of tumor cells to ferroptosis: tumor-suppressor genes reduce cellular antioxidant capacity by transcriptionally inhibiting cystine uptake (56); hypoxic environments remodel lipid metabolism to increase peroxidation substrates (56); the mesenchymal transition state confers cells with unique lipid metabolic features leading to altered susceptibility (57); lipid remodeling directly determines the abundance of oxidizable substrates among membrane phospholipids (58); and membrane repair systems can delay the execution of ferroptosis by patching membrane damage in the final stages of cell death (59). These factors are intertwined, collectively constituting a complex system regulating ferroptosis sensitivity.

## 7 The role of ferroptosis in hepatocellular carcinoma

**Figure 3:**
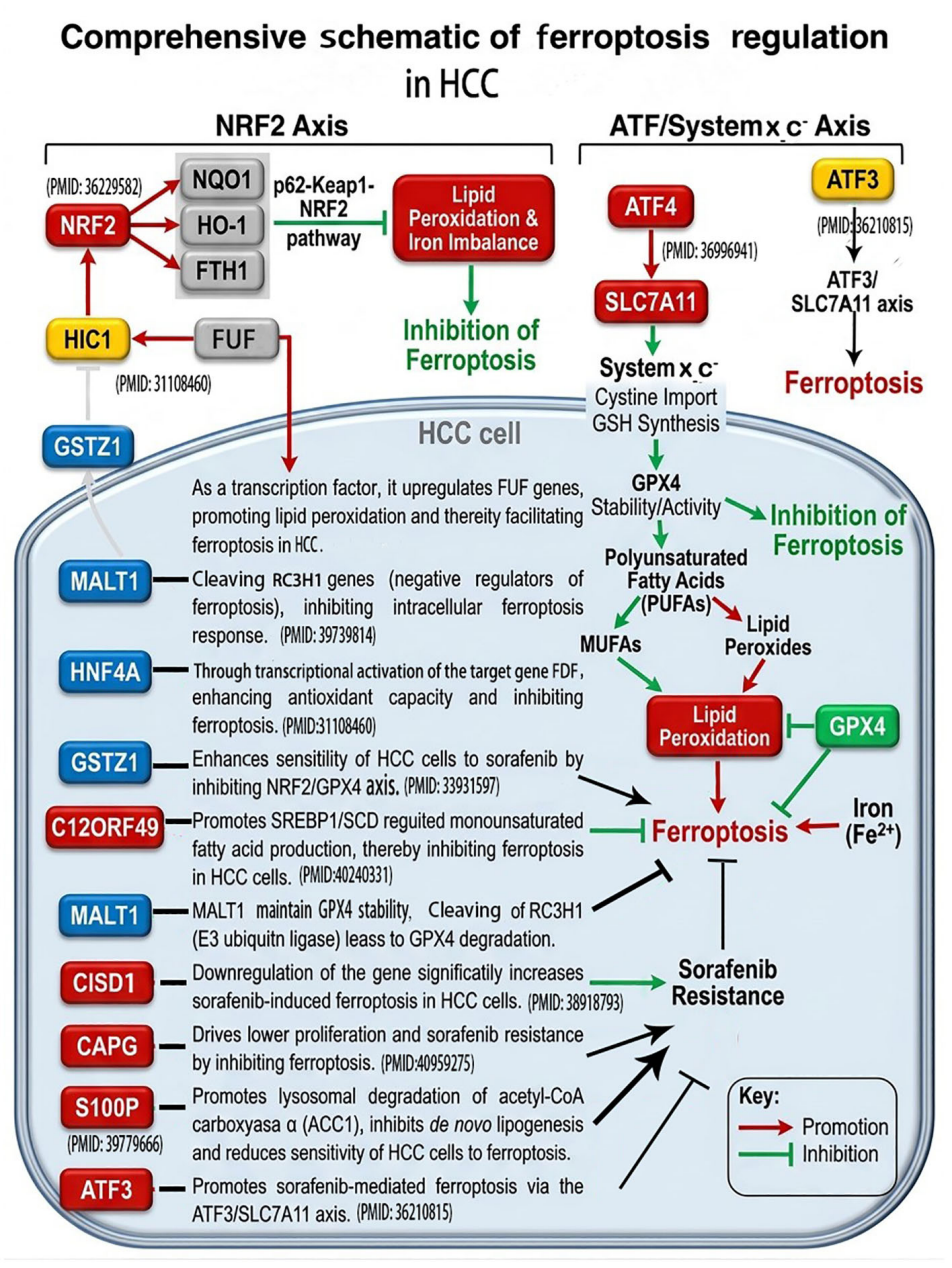
Comprehensive schematic of ferroptosis regulation in HCC. Ferroptosis is driven by lipid peroxidation and iron imbalance and modulated by key regulatory axes: the NRF2 pathway (inhibited by GSTZ1 to enhance sorafenib sensitivity), the ATF/System x_c^–^ axis (where ATF3 regulates SLC7A11 expression), and HNF4A regulate gene FDF expression and GPX4 stability (disrupted by MALT1 and RC3H1). Lipid metabolism critically dictates ferroptosis sensitivity; PUFAs promote ferroptosis, whereas C12ORF49-induced MUFA production and S100P-mediated ACC1 degradation inhibit it. Additionally, CISD1 and CAPG drive sorafenib resistance by inhibiting ferroptosis, while ATF3 promotes sorafenib-induced ferroptosis via the ATF3/SLC7A11 axis.

Hepatocellular carcinoma (HCC) is the most common type of primary hepatocellular carcinoma. It remains a significant challenge in the medical field due to its low detection rate and high mortality. The pathophysiology of hepatocellular carcinoma involves complex signaling pathways that have not yet been fully elucidated. Recent studies have revealed that ferroptosis induced by certain proteins and genes can effectively lead to hepatoma cell death (**Figure 3**). Herein, we focus on several well-established targets. In summary, the unique metabolic vulnerabilities and aberrant signaling pathways that trigger ferroptosis in hepatoma cells hold promise as a prospective therapeutic strategy for hepatocellular carcinoma.

### 7.1 Pro-ferroptosis factors

Activating transcription factor 3 (ATF3) is a classical pro-ferroptosis factor that can be activated by various cellular stressful responses, including DNA damage, oxidative stress, cellular injury (60–63), cell cycle regulation (64), and immune response (65). ATF3 is an endogenous inhibitor of SLC7A11 (63) (SLC7A11 is a core component of the GSH/GPX4 axis; for details, see Section 1.1). In hepatocellular carcinoma (HCC), the ATF3/SLC7A11 axis promotes sorafenib-mediated ferroptosis (61). Similarly, in endometriosis, decreased ATF3 expression leads to increased SLC7A11 transcription, thereby inhibiting ferroptosis (66). However, studies have also indicated that ATF3 is broadly activated by factors such as DNA damage and oxidative stress. Upregulating ATF3 to promote ferroptosis may readily induce off-target effects in normal tissues, leading to ferroptosis damage in cells highly sensitive to oxidative stress, such as intestinal epithelial cells or neurons (67). Therefore, the rational application of ferroptosis for cancer suppression, with due consideration for safety, should be a key focus of attention.

In previous studies, human mucosa-associated lymphoid tissue lymphoma translocation protein 1 (MALT1) was identified as a profound tumor immune evasion factor. It binds to BCL10 via its proteolytic domain to form the CARD–BCL10–MALT1 complex (68), thereby constitutively activating the expression of nuclear factor-κB (NF-κB) target genes, promoting tumor cell survival, proliferation, and metastasis (69). Recent research indicates that MALT1 can regulate the stability of glutathione peroxidase 4 (GPX4) by cleaving the E3 ubiquitin ligase RC3H1 (GPX4 is a core component of the GSH/GPX4 axis; for details, see Section 1.1). Targeting MALT1 can induce ferroptosis in hepatoma cells (70). The novel concept of targeted promotion of immunotherapy for solid tumors has now entered clinical evaluation (71), yet its application in ferroptosis remains relatively underexplored. Additionally, the potential immune imbalance that may arise remains an issue to be addressed (72).

### 7.2 Inhibitory regulatory nodes

S100P is a small calcium-binding protein belonging to the S100 protein family, composed of 95 amino acids (73, 74). In cancer cells such as HCC, S100P is highly expressed and associated with tumor cell growth, invasion, and metastasis, thus often defined as an oncogenic factor (75–77). Studies by Min Yang, Weiwei Cui, and colleagues found that S100P can degrade acetyl-CoA carboxylase α in lysosomes, thereby inhibiting *de novo* lipid synthesis and reducing the sensitivity of hepatoma cells to ferroptosis (78).

The p62–KEAP1–NRF2 axis is also a classical ferroptosis-suppressive pathway. p62, composed of 440 amino acids, regulates various cellular functions (79), acts as a receptor involved in the autophagy of ubiquitinated proteins (80), and is also an antioxidant stress factor. Both NRF2 and its endogenous inhibitor KEAP1 function to defend against cellular oxidative stress (81) (NRF2 can regulate SLC7A11, a core component of the GSH/GPX4 axis; for details, see Section 1.1). Under oxidative stress, cysteine residues in KEAP1 are modified, leading to a conformational change in KEAP1 and inhibition of NRF2 degradation (82). In hepatoma cells, p62 expression can also inhibit NRF2 degradation by inactivating Kelch-like ECH-associated protein 1 (KEAP1), thereby suppressing ferroptosis (83). Here, we summarize the occurrence of ferroptosis driven by distinct mechanistic origins (**Table 1**).

**Table 1:** Summary table of mechanistic sources.

Sources	Mechanisms	Ferroptosis regulatory direction	References	Whether each mechanism directly validated in HCC
Hepatocellular carcinoma	NEAT1–MIOX–ROS	Promotion	21	YES
	ECM–integrin β1/FAK/YAP–ACSL4	Promotion	32	YES
	ATF3–SLC7A11	Promotion	61	YES
	MALT1–RC3H1–GPX4	Inhibition	70	YES
	S100P–ACCα	Inhibition	78	YES
	p62–KEAP1–NRF2	Inhibition	83	YES
Aortic dissection	METTL3–FSP1	Promotion	24	NO
Lung cancer	YTHDC1–FSP1	Promotion	25	NO
Colorectal cancer	FTO–FSP1	Promotion	26	NO
Endometriosis	ATF3–SLC7A11	Promotion	66	NO
Hypotheses require validation	NRF2–SLC7A11–GSH	Inhibition	82	NO

## 8 Advances and challenges in therapeutic translation

**Figure 4:**
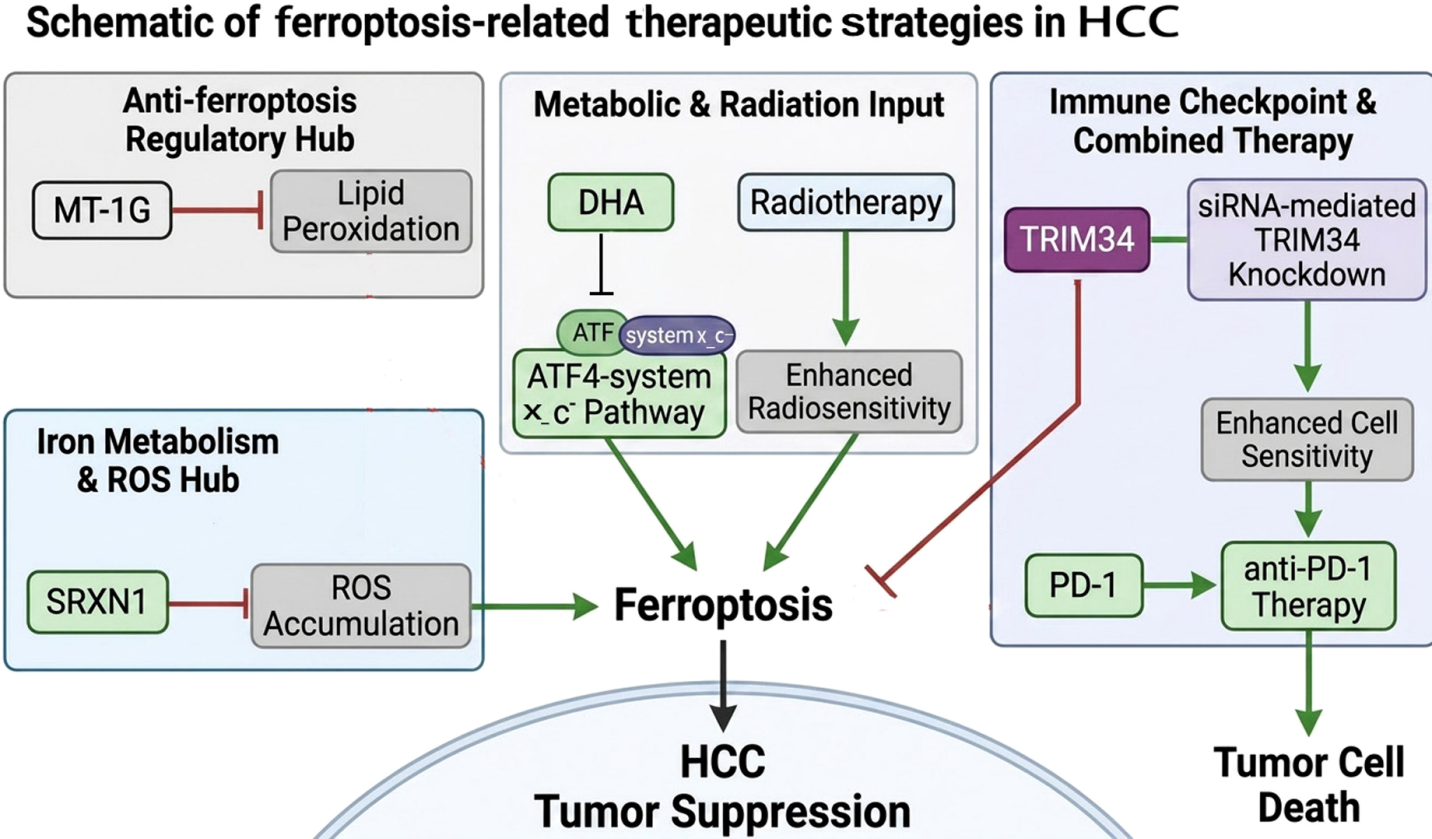
Schematic of ferroptosis-related therapeutic strategies in HCC. Interventions are categorized into three modules: the anti-ferroptosis regulatory hub (targeting MT-1G and the SRXN1 pathway), metabolic and radiation inputs (induces ferroptosis via DHA and radiotherapy), and immune checkpoint combined therapy (anti-PD-1). Collectively, these strategies enhance tumor cell sensitivity and radiosensitivity, ultimately driving ferroptosis-mediated cell death and tumor suppression.

The regulatory nodes in ferroptosis metabolism, epigenetics, and the microenvironment of hepatocellular carcinoma (HCC) discussed above all provide potential therapeutic targets. Monotherapy and combination therapy strategies based on these targets offer new directions for overcoming resistance to conventional HCC treatments. However, attention must also be paid to the toxicity risks during treatment and the challenges of clinical implementation. Therefore, this review summarizes the therapeutic strategies related to ferroptosis in HCC (**Figure 4**).

### 8.1 Monotherapy strategies

Since its approval in 2007, sorafenib has remained the cornerstone of treatment for advanced HCC. In the 2021 NCCN guidelines, sorafenib is no longer the default first choice for Child–Pugh A (84). Immune-based combination therapy has become an important first-line option for eligible patients. For example, atezolizumab and bevacizumab immunotherapy combination has become the guideline-recommended first-choice regimen. Also, clinical evidence indicates that many HCC patients cannot tolerate the toxic side effects of sorafenib, and some develop adaptive resistance (85). Consequently, research into reversing sorafenib resistance is urgently needed. Metallothionein-1G (MT-1G) is a key target in sorafenib resistance. Preclinical ferroptosis strategy studies have found that upregulating MT-1G inhibits lipid peroxidation levels in HCC. Blocking its expression can induce ferroptosis, thereby enhancing the antitumor efficacy of sorafenib (86). Sulfiredoxin-1 (SRXN1) is an endogenous antioxidant enzyme that is highly expressed in HCC tissues and promotes HCC cell proliferation. Inhibiting SRXN1 leads to reactive oxygen species (ROS) accumulation (87). Thus, inhibiting MT-1G and SRXN1 is considered a promising monotherapy strategy to overcome sorafenib resistance by restoring ferroptosis.

The potential of natural compounds as monotherapies is also gaining attention. Dihydroartemisinin (DHA), a derivative of artemisinin, exhibits significant anticancer mechanisms, including inhibiting tumor metastasis and inducing tumor cell apoptosis (88). Preclinical proof-of-concept studies have shown that in HCC, DHA induces ferroptosis by inhibiting the ATF4- system x_c^–^ pathway, thereby enhancing the chemosensitivity of HCC to sorafenib both *in vivo* and *ex vivo* (89). This demonstrates its potential for development as a clinical monotherapy.

### 8.2 Combination therapy strategies

The combination of immunotherapy and molecular targeted therapy has achieved encouraging results in cancer therapies and represents a potential new treatment avenue. Anti-programmed cell death protein 1 (PD-1) immunotherapy aims to achieve antitumor effects by using PD-1 monoclonal antibodies to block the binding of PD-1 to its ligands (90). However, anti-PD-1 therapy often fails due to the absence or low expression of the ligand PD-L1, lack of immune cells, and other reasons (91). Studies have found that in HCC, knocking down the expression of the ferroptosis suppressor TRIM34 improves the response to anti-PD-1 therapy (92). Furthermore, inhibiting phosphoglycerate mutase 1 (PGAM1) exerts antitumor effects by promoting ferroptosis and CD8^+^ T-cell infiltration and also exhibits synergistic effects with anti-PD-1 immunotherapy in HCC (93).

Radiotherapy is a common way to treat cancer, but dealing with resistance and side effects is still a challenge. Research has found that ionizing radiation in radiotherapy can effectively kill tumors by promoting cellular ferroptosis (94). Pro-ferroptosis target genes, such as collectrin (CLTRN) and the copper metabolism MURR1 domain-containing protein 10, can enhance radiosensitivity and may become new targets for HCC treatment (95, 96).

### 8.3 Toxicological risks of ferroptosis therapy

Although ferroptosis demonstrates significant anticancer potential in hepatocellular carcinoma (HCC), its reliance on iron ions and reactive oxygen species (ROS) for cell death also poses substantial risks of systemic toxicity. A review summarized the side effects of ferroptosis-based antitumor therapy from five aspects (97): (1) immunosuppression—oxidized lipids released by tumor cells undergoing ferroptosis can inhibit T-cell function, thereby weakening the body’s antitumor immune surveillance; (2) visceral organ iron overload—the liver and kidneys, being key organs for iron storage and excretion, are susceptible to ferroptosis-related toxicity; (3) bone marrow suppression—damage to hematopoietic stem cells can lead to anemia and decreased immunity; (4) cachexia—resulting in weight loss and muscle atrophy; (5) subsequent tumors—potential induction of mutations in surrounding normal tissues. Studies have also found that ferroptosis inducers can promote tumor formation in pancreatic ductal adenocarcinoma (PDAC) (98). However, research on the differential manifestations and toxicological risks of ferroptosis across various HCC subtypes remains lacking.

### 8.4 Clinical challenges

Despite the well-defined target mechanisms of therapeutic strategies, translating these findings into clinical efficacy faces significant obstacles. Monotherapy strategies are often based on high expression profiles observed in ex vivo cell lines or animal models. Clinically, however, the high degree of tumor heterogeneity in HCC patients makes it difficult to screen for patients with high expression of MT-1G or SRXN1. Natural compounds have a short half-life, are rapidly metabolized, and require high concentrations for efficacy, which increases the risk of toxicity (85). Combination therapy strategies also face multiple challenges in transitioning from “proof-of-concept” to clinical application. Although PD-1/PD-L1 antibodies show promise, immune-related adverse events remain a critical clinical issue. Concurrently, ferroptosis inducers often lack sufficient selectivity between normal cells and tumor cells, potentially leading to severe side effects. Radiotherapy may disrupt T cells within the tumor microenvironment, diminishing the efficacy of immunotherapy; optimizing the timing window between radiotherapy and immunotherapy is therefore crucial.

The occurrence of hepatocellular carcinoma (HCC) is often accompanied by chronic liver injury and fibrosis (99). During the early stage of liver injury, ferroptosis of hepatocytes is a key driver of inflammation and fibrosis (100), whereas after HCC tumorigenesis, ferroptosis becomes a crucial mechanism for eliminating tumor cells (101). Most existing studies focus on “how to induce ferroptosis in HCC” yet rarely evaluate the lethal impact of systemically induced ferroptosis on normal/Para cancerous hepatocytes within the context of cirrhosis. Forcibly activating ferroptosis may trigger fulminant hepatic failure (102). The “therapeutic window” for ferroptosis-targeted therapy in HCC is extremely narrow (103), and current preclinical studies lack a profound consideration of the temporal differences across various stages of liver disease.

Early screening and efficacy monitoring for HCC still primarily rely on alpha-fetoprotein (AFP), des-gamma-carboxy prothrombin (DCP), AFP-L3, and combinations of several microRNAs (miRNAs). However, the sensitivity of these markers in early-stage (I/II) disease remains insufficient, with diagnostic rates declining significantly, particularly in AFP-negative patients. Consequently, there is an urgent need to supplement these with indicators that can reflect tumor metabolism, oxidative stress, or iron metabolism abnormalities. 4-Hydroxynonenal (4-HNE) is currently a promising multi-marker panel candidate. Studies indicate that 4-HNE levels increase in response to the degree of ferroptosis, and in hepatoma cells, low levels are associated with more aggressive tumor behavior (104). Furthermore, commercially available competitive enzyme-linked immunosorbent assay (ELISA) kits provide a technical foundation for translating 4-HNE into a clinical detection tool for HCC. However, 4-HNE is not a specific biomarker for HCC, as its levels are influenced by extensive lipid peroxidation and oxidative stress. Studies have demonstrated that 4-HNE levels are significantly elevated in patients with chronic liver diseases such as non-alcoholic steatohepatitis (NASH) and hepatitis B/C (105, 106). Serum/plasma is most used in clinical screening but heavily affected by overall inflammation. ELISA is easy to operate and low cost but specificity depends on antibody quality and is easily affected by cross-reactions. In liver disease studies, 4-HNE mostly shows how much lipid peroxidation and oxidative stress is going on. You can think of it as a marker of oxidative stress, and its level changes more so show how well the body is defending itself against oxidative stress. While 4-HNE exhibits certain prognostic value in hepatocellular carcinoma (HCC), its potential as an auxiliary biomarker for hepatocellular carcinoma warrants further exploration.

HCC treatment involves surgery, local ablation, Trans arterial interventions (Trans arterial chemoembolization/hepatic arterial infusion chemotherapy, TACE/HAIC), and systemic targeted/immunotherapy. Different treatment modalities carry varying degrees of toxicological risk to the liver, kidneys, heart, and hematopoietic system, necessitating systematic monitoring and intervention throughout the treatment course. Currently, specific reversal agents for ferroptosis are lacking. Once severe ferroptosis toxicity occurs, clinical intervention becomes extremely challenging.

## 9 Conclusion

Although the theoretical framework of ferroptosis for the treatment of HCC has been extensively developed, it remains largely confined to preclinical studies, with no specific clinically approved drugs available to date. Although the involvement of ferroptosis mechanisms has been demonstrated in cellular and animal experiments for HCC therapeutic agents such as sorafenib (85), they are not classified as ferroptosis-specific drugs. Various combination treatment plans have shown efficacy in animal studies, but data from phase I/II clinical trials are lacking. Although 4-HNE has been proposed as a potential biomarker (104), its clinical specificity has not been validated in large-scale cohorts. Furthermore, evidence for certain mechanisms in HCC remains insufficient, primarily based on analogical inferences from other cancer types.

Ferroptosis in HCC is not an isolated event but rather a self-reinforcing network driven by the interplay of iron metabolism imbalance, lipid peroxidation, and defective antioxidant defenses. The positive feedback loop between reactive oxygen species (ROS) and ferrous ions constitutes its core dynamic. Microenvironmental factors, including non-coding RNAs, posttranscriptional RNA modifications, and extracellular matrix (ECM) stiffness, directly determine the sensitivity of cancer cells to ferroptosis by regulating the expression of key enzymes. Although ferroptosis inducers have demonstrated potential to overcome sorafenib resistance, their systemic toxicity (including kidney damage and liver damage, immunosuppression) and heterogeneous performance within HCC represent the most significant current bottlenecks. Therefore, further in-depth research is required in the following two directions to deepen our understanding of ferroptosis mechanisms and their application in HCC therapy.

Deepening of mechanistic studies: In-depth analysis is needed to obtain direct evidence for ROS-induced DNA damage and TET demethylase inhibition triggered by iron overload. The specific mechanism by which IFN-γ inhibits GPX4 through the downregulation of system x_c^–^ expression and reduces GSH synthesis in HCC requires clarification.

Potential breakthroughs for clinical translation: The clinical value of oxidative stress product 4-HNE as a potential biomarker for early diagnosis and efficacy monitoring in HCC needs validation. Establishing an HCC Safety Monitoring Panel, focusing on liver and kidney function, myelosuppression, and immune function, is crucial for improving treatment safety and prolonging patient survival.
